# Inflammation and structural changes of splenic lymphoid tissue in visceral leishmaniasis: A study on naturally infected dogs

**DOI:** 10.1111/j.1365-3024.2008.01051.x

**Published:** 2008-10

**Authors:** C C Santana, J Vassallo, L A R De Freitas, G G S Oliveira, L C Pontes-De-Carvalho, W L C Dos-Santos

**Affiliations:** 1 Centro de Pesquisas Gonçalo Moniz, Fundação Oswaldo Cruz, Fundação Oswaldo CruzCandeal, Salvador, Bahia, Brazil; 2 Faculdade de Ciências Médicas, Universidade de Campinas, Cidade Universitária ‘Zeferino Vaz’Campinas, São Paulo, Brazil

**Keywords:** *canine visceral leishmaniasis*, Leishmania chagasi, Leishmania infantum, *lymphoid tissue*, *Spleen*

## Abstract

The aim of this study was to identify splenic immuno-inflammatory patterns associated with natural infection by *Leishmania chagasi*. Spleen samples were obtained from 72 stray dogs from an endemic area of visceral leishmaniasis. The animals were grouped into four categories as follows: (i) potentially resistant to visceral leishmaniasis, with a positive leishmanin skin test result, and negative splenic culture for *Leishmania* parasites (ii) potentially susceptible to visceral leishmaniasis, with a negative leishmanin skin test and positive splenic culture for *Leishmania* (iii) infected with undefined susceptibility status, with a positive leishmanin skin test and positive splenic culture for *Leishmania*, and (iv) noninfected, with a negative leishmanin skin test, negative splenic culture for *Leishmania*, and negative serology for anti-*Leishmania* antibodies. Histopathological analyses showed that there was a higher frequency of perisplenitis (18/25, *P* < 0·0001), granuloma (7/25, *P* = 0·0102), structural disorganization (14/25, *P* < 0·0001), and atrophy of the lymphoid follicles (20/25, *P* = 0·0036) and of the marginal zone (15/25, *P* = 0·0025) in the potentially susceptible group than in the other groups. The data presented here show changes in the white pulp of the spleen that are associated with naturally acquired visceral leishmaniasis.

## Introduction

Visceral leishmaniasis is used to describe two clinically, parasitologically and epidemiologically different diseases: antroponotic visceral leishmaniasis, caused by *Leishmania donovani*, and zoonotic visceral leishmaniasis (VL), caused by *L. chagasi*/*L. infantum*. The latter disease is endemic in Mediterranean countries and the American continent, and its causal agents are transmitted to mammalian hosts by sand flies of the *Phlebotomus* and *Lutzomyia* genera (1). *Leishmania chagasi*/*L. infantum* infection does not always lead to VL (2). Most infected humans (2,3) and at least a proportion of infected dogs (4,5) remain asymptomatic or develop a mild disease, which is spontaneously cured. Occasionally, dogs and humans develop a severe form of VL (2,6) that is usually lethal if left untreated. In this report, we use the term VL to refer to this severe form of the disease. In both humans and dogs, the disease proceeds with emaciation, enlargement of the liver and spleen, fever, anaemia, and an increased predisposition to bacterial infection (2,7–10). In dogs, such signs of disease are further accompanied by a variety of skin and ocular lesions (9). Much that is known about the immune response to *Leishmania* parasites, and even the immune response in general has come from studies in murine models of leishmaniases (11–13). Most of these models, however, are intrinsically compromised by the artificial nature of the infective inocula. Instead of consisting only of naturally infective, metacyclic promastigotes, these inocula usually contain (i) either relatively large number of complement-fixing, noninfective procyclic promastigotes (usually constituting more than 70% of the inoculum) (14) or (ii) amastigotes, which are not the usual infectious form of the parasite. Canine VL, arising from natural infection by *L. chagasi*, is a disease worthy not only of study in its own right for veterinary and epidemiological reasons (15), but also for consideration as an attractive animal model for the human disease, because the infective inoculum is very likely identical to that causing human infection in endemic areas. Observations made in canine VL may therefore confirm and refine conclusions drawn from studies using murine experimental models.

Recently, we observed that a positive leishmanin skin test (LST) was negatively associated with emaciation and specific antibody activity *in Leishmania*-infected dogs (16). In the present study, we examined the spleen histology of these animals to identify alterations potentially associated with disturbances in the immune response to *Leishmania* and other pathogens that occur at late stages of the disease.

We decided to examine the spleen because it plays a central role in VL. The spleen is infected in all cases of the disease. In contrast to other organs like the liver, the spleen maintains the infection during the entire course of VL (11,12). In fact, a splenic index of parasitism is used as a clinical criterion of therapeutic response in human VL (17). The spleen is also a lymphoid organ with cellular diversity and compartmentalized microenvironments, suitable for the definition of immunologic processes that may influence the outcome of infections (18,19). During the course of VL, the spleen becomes an evident site of interaction between the immune system and the *Leishmania*, because all of the obligatory participants in the immune response against the parasite are present in large quantities. These include the antigen (live parasites or their debris), antigen-presenting cells, and lymphocytes capable of responding to these antigens. Changes in the splenic microenvironment have been identified following the experimental infection of mice by a variety of pathogens, including *Leishmania* (20,21). Our hypothesis is that such alterations are also present in the course of natural infection by *L. chagasi* in dogs and that they reflect a status of susceptibility to the disease.

## Materials and Methods

### Animals

Spleen samples from 72 dogs were used in this study. The specimens were obtained from the archive of histological samples of the Gonçalo Moniz Research Center, FIOCRUZ, Salvador, Brazil. These animals were stray dogs of different breeds and estimated ages collected from the streets of Jequié (Bahia State, Brazil, an endemic area for visceral leishmaniasis) at different periods between 1998 and 2001 in collaboration with the Endemic Diseases Surveillance Program of the State Health Service. The nutritional status of the animals was recorded into two categories: showing evidence of emaciation (when prominences of bones from the vertebral column, iliac crest and ribs were present), or normal (when bone prominences were absent). The presence of anti-*Leishmania* antibodies in the serum were investigated by ELISA, and a cellular immune response against *L. chagasi* antigens was detected by LST. The details of these tests are reported elsewhere (16,22,23). The animals were kept for at least 48 h in the municipality kennel. When not claimed by their owners, the animals were culled, as recommended by the Brazilian program for the control of zoonotic diseases. Spleen specimens were then collected for both *Leishmania,* detection by *in vitro* culture, and histology. All procedures were conducted in accordance with the Oswaldo Cruz Foundation guidelines for animal experimentation. All of the animals that had detectable *Leishmania* in their spleen cultures or a positive LST, with available spleen samples, were included in the study. The animals were distributed into the following categories: (i) Infected and potentially resistant to VL – 22 animals with a positive LST and negative spleen culture for *Leishmania*; (ii) Infected and potentially susceptible to VL – 25 animals with a negative LST and positive spleen culture for *Leishmania*; (c) Infected with undefined susceptibility status –11 animals with both a positive LST and positive spleen culture for *Leishmania*. As a control group, specimens from 14 animals with negative spleen cultures, a negative LST, and the absence of anti-*Leishmania* antibody activity in the serum as measured by ELISA were randomly selected; we referred to this group as noninfected. Table 1 summarizes the characteristics of the groups used in the study.

**Table 1:** General characteristics of a sample of dogs, from an endemic area of visceral leishmaniasis, used in this study

	Groups of animals			
		
	Infected			
		
Parameters	Potentially Resistant to VL	Potentially susceptible to VL	Undefined susceptibility status	Noninfected
Number of animals	22	25	11	14
Spleen culture	–	+	+	–
Leishmanin’ skin test	+	–	+	–
Serology^a^	0·55 ± 0·39	1·54 ± 0·91^b^	0·87 ± 0·77	0·28 ± 0·13
With emaciation^c^	6/20 (30%)	15/25 (60%)^d^	2/11 (18%)	4/13 (31%)

a Expressed as mean ± SD of the OD. 490 nm obtained in indirect ELISA for detection of anti-*Leishmania* antibodies in the serum;

b Statistically significant from the control group, Kruskal–Wallis test, *P* < 0·001;

c Number of emaciated animals/total number of animals in which emaciation was investigated (%);

d Statistically significant from the other groups, χ^2^-test *P* = 0·02.

### Spleen samples and histological analysis

**Figure 1:**
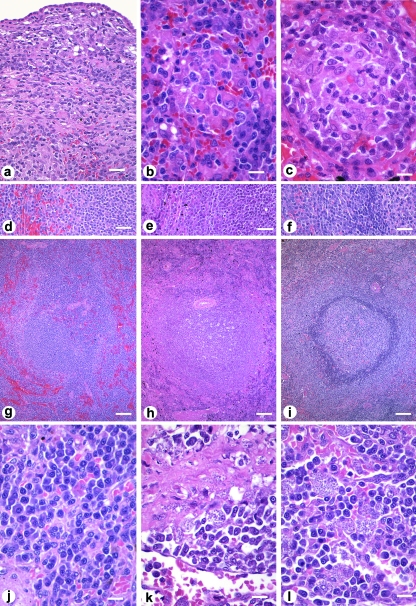
Morphological changes in the spleen of dogs naturally infected by *L. chagasi*. (a) Chronic perisplenitis; (b) Ill-structured granuloma with a loose grouping of a few epithelioid macrophages; (c) Well-structured granuloma with tightly grouped epithelioid macrophages in a round structure rimmed by lymphocytes and plasma cells; (d)–(i) Architectural definition of the lymphoid follicles (g) extensively disorganized lymphoid tissue; (h) moderately disorganized lymphoid tissue; (i) well organized lymphoid tissue (d, e, and f – details of the follicular borders); (j) Increased plasma cell number in the red pulp; (k) Parasite-loaded macrophages in the subcapsular area; (l) Parasite-loaded macrophages in the internal red pulp. Lengths of the scale bars on the right hand side at the bottom of the figure: a, 50 µm; b, c, j, k and l, 25 µm; d, e and f, 100 µm; g, h and i, 250 µm.

Three to 4 mm-thick slices of spleen tissue were cut transversally to the capsule and fixed in 10% formalin. After fixation, tissue slices were embedded in paraffin. Four to 5 µm-thick sections were cut and stained with haematoxylin and eosin (H&E). These sections were examined by at least two of the authors who were blind to previous knowledge of the identities of the animals. The authors observed the following parameters:

1. *Presence of perisplenitis*, as evidenced by a focal thickening of the splenic capsule with an inflammatory infiltrate frequently associated with incipient fibrosis (Figure 1a).
2. *Presence of granuloma*, with focal aggregates of four or more epithelioid macrophages (Figure 1b,c).
3. *Lymphoid follicle frequency*, in which the density of lymphoid follicles in the splenic tissue sections was classified as rare (follicles were infrequently found), low (follicles were easily found but sparse in the red pulp), average (follicles were uniformly distributed and easily found in the section) and high (follicles were frequent and large).
4. *Size of lymphoid follicles, germinal centre and marginal zone,* in which the lymphoid follicles and the marginal zone were classified as tiny, small, average and large.
5. *Degree of structural organization of the white pulp*, in which the white pulp was classified as: *well organized*, with distinct peri-arteriolar lymphocyte sheath, germinal centre, mantle zone and marginal zone (24); *slightly disorganized*, with either hyperplastic or hypoplastic changes leading to a loss in definition of any of the regions of the white pulp; *moderately disorganized*, when the white pulp was evident, but its regions were poorly individualized or indistinct; *extensively disorganized*, when the follicular structure was barely distinct from the red pulp and T-cell areas (Figure 1d–i). The last two categories were frequently associated with lymphoid atrophy.
6. *Frequencies of cell populations in the red pulp*, with the frequencies of lymphoblasts, plasma cells, macrophages, granulocytes, and megakaryocytes semiquantified as absent, rare (seen as isolated cells or small aggregates in a small proportion of the examined ×400-magnified microscopic fields), few (seen as isolated cells or small aggregates in approximately half of the examined ×400-magnified microscopic fields), average (seen as single cells or aggregates observed in most of the examined ×400-magnified microscopic fields), or frequent (consisting of single cells or aggregates seen in roughly all of the examined ×400-magnified microscopic fields). The cells were characterized morphologically according to the following criteria:
  - Lymphoblasts: Cells with round nuclei containing condensed chromatin, inconspicuous nucleoli, and scant cytoplasm.
  - Plasma cells: Cells with eccentric nuclei, heterochromatin dispersed around the edge in a pattern similar to the numerals of an analogue clock face, and basophilic cytoplasm with clear, perinuclear vacuoles.
  - Macrophages: Large cells with oval or reniform nuclei containing loosely packed chromatin surrounded by a rim of eosinophilic cytoplasm with indistinct edges.
  - Granulocytes: Cells with highly lobulated nuclei with densely packed chromatin.
  - Megakaryocytes: Very large cells with wide and clearly defined cytoplasm and large, lobulated nuclei containing condensed chromatin.
7. *Parasite burden*, where the amount of amastigotes in the spleen sections was estimated by conventional light microscopy examination of a minimum of 40 and a maximum of 100 nonoverlapping high-power (×1000-magnified) fields per section. The examined fields were equally distributed between the subcapsular (Figure 1k) and internal red pulp (Figure 1l). The results are expressed as the proportion of independent fields with amastigotes.

### Expression and analysis of the results

The numerical data shown in the text, table and graphs represent absolute or relative numbers of animals with the corresponding condition, unless otherwise stated. The significance of the differences between the proportions of the studied variables among the groups was tested using the χ^2^-test with a Yates’ correction, or a Fishers’ exact probability test when recommended. When appropriate, a paired *t*-test or the Kruskal–Wallis statistic were used for testing differences involving the means of numerical data obtained from different groups (25). The critical level of significance was set at *P* < 0·05%.

## Results

### Perisplenitis

Perisplenitis was present in 20 (28%) out of 72 animals (Table 2 and Figure 1a). It was more frequent in animals in the infected and potentially susceptible to VL group (18 out of 25 animals, 72%) than other groups (χ^2^-test, *P* < 0·0001).

**Table 2:** Perisplenits and red pulp changes in dogs from an endemic area of *L. chagasi* infection, with different patterns of response to the infection

	Animal groups				
			
	Infected				
			
Parameters	Potentially resistant to VL (%)	Potentially susceptible to VL (%)	Undefined susceptibility status (%)	Noninfected (%)	Total (%)
Number of animals	22	25	11	14	72
Perisplenitis	2 (10)^a^	18 (72)^b^	0 (0)	0 (0)^a^	20 (28)
Red Pulp:					
Granuloma	0 (0)	7 (28)^c^	1 (9)	0 (0)	8 (11)
Plasma cells:					
Absent–few	5 (23)	1 (4)	2 (18)	6 (43) d	14 (19)
Average–frequent	17 (77)	24 (96)	9 (82)	8 (57)	58 (81)
Macrophage:					
Absent–few	13 (59)	15 (60)	0 (0)	4 (29)	32 (44)
Average–frequent	9 (41)	10 (40)	11 (100)	10 (71)	40 (55)
Lymphoblasts:					
Absent-few	19 (86)	17 (68)	9 (82)	9 (64)	54 (75)
Average-frequent	3 (14)	8 (32)	2 (18)	5 (36)	18 (25)
Megakaryocyte:					
Absent-few	14 (64)	19 (76)	8 (72)	12 (86)	53 (74)
Average-frequent	8 (36)	6 (24)	3 (27)	2 (14)	19 (26)
*Leishmania amastigotes*:	0 (0)	10 (40)^e^	0 (0)	0 (0)	10 (14)
Positive histology:					
Sub-capsular	0 (0)^g^	10 (40)^e^	0 (0)	0 (0)	10 (14)
Red pulp	0 (0)	10 (40)^e^	0 (0)	0 (0)	10 (14)
Parasite density:					
Sub-capsular	0	45 ± 40^f^,^g^	0	0	
Internal red pulp	0	37 ± 44	0	0	

^a^Capsular tissue was absent or poorly represented in one animal. ^b–f^ Statistically significant differences (χ^2^-test with Yates’ correction):

a *P* = 0.02;

b different from the other groups, *P* < 0.0001;

c different from the other groups, *P* = 0.0102;

d different from the other groups, *P* < 0·0366;

e different from the other groups, *P* < 0.0001;

f Statistically different from the parasite density in the internal red pulp (paired *t*-test, *P* = 0.0425).

g Expressed as the mean of the relative (%) number of high power fields positive for amastigotes among 20–50 examined.

### Granuloma

Poorly structured aggregates, consisting of at least four large epithelioid macrophages (Figure 1b), to slightly organized granulomas (Figure 1c) were observed in 8 (11%) out of 72 animals (Table 2 and Figure 1b). They were present in 1 (9%) out of 11 animals in the undefined susceptibility state group and 7 (28%) out of 25 animals in the infected and potentially susceptible to VL group. The difference in the presence of spleen granulomas between the infected and potentially susceptible to VL and other groups was statistically significant (χ^2^-test, *P* = 0·0102).

### Cell populations in the red pulp

The frequency of plasma cells was higher in the groups with evidence of *Leishmania* infection than it was in the noninfected group (χ^2^-test, *P* = 0·0366, Table 2 and Figure 1j). The distribution of lymphoblasts, granulocytes, resident macrophages and megakaryocytes did not differ significantly among the groups (Table 2).

### Parasite burden

Amastigotes were visualized in the spleens of 10 (14%) out of the 72 animals (Table 2); all of these animals belonged to the infected and potentially susceptible to VL group. In all of these animals, amastigotes were seen in both the subcapsular area and the internal red pulp (Figure 1j–k). The parasite burden, measured as the proportion of positive fields among the examined high power fields, was higher in the subcapsular area (45 *±* 40%) than the internal red pulp (37 + 44%; paired *t*-test, *P* = 0·0425). Although parasite distribution in the spleen was usually uneven, three animals with high parasite burden showed evidence of parasites in all of the examined fields (i.e. in the subcapsular area and in the internal red pulp).

### White pulp structural organization

Distinction of the different regions inside the white pulp of the spleen was absent or poor in 17 (24%) out of 72 animals (Table 3 and Figure 1d–i). The frequency of animals with moderate or extensive structural disorganization of the white pulp areas was higher in the infected and potentially susceptible to VL group (14 out of 25 animals, 56%) than it was in the other groups (χ^2^-test, *P* < 0·0001).

**Table 3:** White pulp changes in dogs from an endemic area of *L. chagasi* infection, with different patterns of response to the infection

	Animal groups				
			
	Infected				
			
Parameters	Potentially resistant to VL (%)	Potentially susceptible to VL (%)	Undefined susceptibility status (%)	Noninfected (%)	Total (%)
Number of animals	22	25	11	14	72
White Pulp					
Structural disorganization:					
Absent – Slight	21 (95)	11 (44)	10 (91)	13 (93)	55 (76)
Moderate – Extensive	1 (5)	14 (56)^a^	1 (9)	1 (7)	17 (24)
Lymphoid follicle:					
Frequency					
Rare – Low	6 (27)	20 (80)^b^	2 (18)	8 (57)	36 (50)
Average – High	16 (73)	5 (20)	9 (82)	6 (43)	36 (50)
Size					
Tiny – Small	6 (27)	20 (80)^c^	4 (36)	8 (57)	38 (53)
Average – Large	16 (73)^d^	5 (20)	7 (64)	6 (43)	34 (47)
Germinal centre:					
Size					
Absent – Small	6 (27)	21 (84)^e^	5 (45)	7 (50)	39 (54)
Average – Large	16 (73)^f^	4 (16)	6 (55)	7 (40)	33 (46)
Marginal zone:					
Size:					
Absent – Small	5 (23)	15 (60) g	2 (18)	3 (21)	25 (35)
Average – Large	17 (77)	10 (40)	9 (82)	10 (79)	47 (65)

^a–e^Statistically different from the other groups (χ^2^-test with Yates’ correction):

a *P* < 0·0001;

b *P* = 0·0010;

c *P* < 0·0036;

d *P* = 0·044;

e *P* = 0·0015;

f *P* = 0·0162;

g *P* = 0·0025.

### Lymphoid follicle frequency

Lymphoid follicles were present in specimens from all of the animals (Table 3). The frequency of these follicles was rare or low in 36 (50%) out of the 72 animals. The frequency of animals with a low proportion of lymphoid follicles was higher in the infected and potentially susceptible to VL group (20 out of 25, 80%) than it was in the other groups (χ^2^-test, *P* = 0·001).

### Lymphoid follicle size

Lymphoid follicles were tiny or small in 38 (53%) out of the 72 animals (Table 3). A higher proportion of animals with small or tiny lymphoid follicles was observed in the infected and potentially susceptible to VL group (20 out of 25 animals, 80%) than other groups (χ^2^-test, *P* = 0·0036). In contrast, the frequency of animals with large follicles in the infected and potentially resistant to VL group (16 out of 22 animals, 73%) was higher that in the other groups (χ^2^-test, *P* = 0·044).

### Germinal centre size

Germinal centres were absent or small in 39 (54%) out of the 72 animals (Table 3). The frequency of animals with lymphoid follicles containing small, tiny, or the absence of germinal centres was higher in the infected and potentially susceptible to VL group (21 out of 25 animals, 84%) than other animal groups (χ^2^-test, *P* = 0·0015). Conversely, the frequency of animals with average or large germinal centres was higher in the infected and potentially resistant to VL group (16 out of 22 animals, 73%) than the other groups (χ^2^-test, *P* = 0·0162).

### Marginal zone size

**Figure 2:**
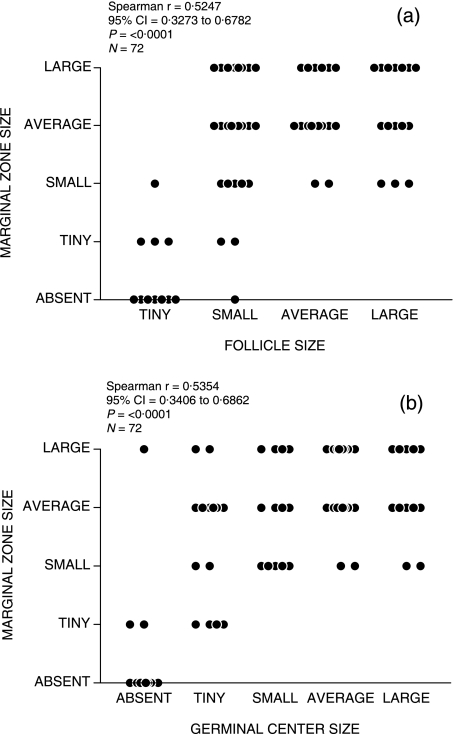
Association between changes in the follicles and marginal zones of the spleen of dogs from an endemic area of visceral leishmaniasis. (a) association between follicle size and marginal zone size; (b) association between germinal centre size and marginal zone size. Each circle on the graph represents data from one animal.

The marginal zone was absent, tiny, or small in 25 (35%) out of the 72 animals (Table 3). The proportion of animals with marginal zones that were small, tiny, or absent was higher in the infected and potentially susceptible to VL (15 out of 25 animals, 60%) than the other animal groups (χ^2^-test, *P* = 0·0025). The marginal zone size was moderately correlated with lymphoid follicle size (Spearman *r* = 0·5247, *P* < 0·0001, Figure 2a) and germinal centre size (Spearman *r* = 0·5354, *P* < 0·0001, Figure 2b).

### Associations between morphological changes in the spleen and emaciation

Evidence of weight loss was present in 27 (39%) out of the 69 animals from which data were recorded. Weight loss was more frequent in the infected and potentially susceptible to VL group than other groups (χ^2^-test, *P* = 0·02). Being underweight was associated with the following alterations in the splenic tissue. First, it was associated with disorganization of the white pulp structure. Among the 27 animals with emaciation, 19 (70%) had some degree of white pulp disorganization; only 18 (43%) out of 42 animals with normal nutritional status had loss of white pulp architecture definition (χ^2^-test, *P* = 0·0467). This association between emaciation and disorganization of the lymphoid tissue was observed even in animals with negative splenic culture. Out of 10, 8 (80%) animals who were undernourished in this group had loss of white pulp architecture definition, whereas only 9 (36%) out of 25 without emaciation exhibited this alteration. In addition, being underweight was associated with perisplenitis. This condition was present in 12 (46%) out of 26 animals with emaciation, but in only 6 (15%) out of 41 animals with normal nutritional status (χ^2^-test, *P* = 0·0107).

## Discussion

Changes in lymphoid organs associated with VL have been previously reported in various studies. However, the current study presents a systematic view of changes in different compartments of the spleen in naturally infected dogs from an endemic area of VL, and describes different patterns of immunological responses to the parasite. In a previous study, we have shown that negative LST results were associated with high levels of serum antibody activity and emaciation in *L. chagasi*-infected dogs. We have now shown that infected animals with negative LST also have (i) increased frequencies of perisplenitis, granuloma, and parasites in the red pulp (ii) a variety of white pulp alterations, such as atrophy and loss of structural definition (iii) lymphoid follicles that are reduced in number and size, and (iv) germinal centres and marginal zones that are frequently small or absent. These animals, with loss of lymphoid follicle definition, perisplenitis, and a high parasite density in the spleen (in which parasites were seen by conventional histology), were also more frequently underweight; this association likely reflects the status of the clinical disease. Furthermore, we observed that the distribution of parasites in the spleen was not homogeneous. In heavily infected animals, parasites were distributed almost continuously in the red pulp. In animals with a less intense parasite burden, a discontinuous distribution of infected cells was observed, predominating in the subcapsular area.

Perisplenitis is a common finding in canine VL (26,27). Although the increase in spleen size may predispose that organ to trauma or mechanical stress, the actual genesis of perisplenitis in leishmaniasis remains unknown. It is interesting, however, that the perisplenitis was associated with the presence of parasite-containing macrophages in subcapsular areas. The presence of such macrophages in capsular and subcapsular inflammatory infiltrates has also been observed in other studies (26,27). These findings may indicate a role for inflammation in parasite dissemination (28,29), or that the parasite elicits an inflammatory reaction.

In light of previously published data regarding immunity to *Leishmania*, it seems contradictory that splenic granulomas were more frequently found in the group of infected animals with negative LST than in infected animals with positive LST. In effect both, granuloma and positive LST, may result from DTH reactions to *Leishmania* antigens. The granulomas observed in the spleen of the animals in this study varied from poorly structured aggregates, consisting of at least four large epithelioid macrophages (Figure 1b), to the barely organized structure shown in Figure 1(c). Although parasites were not usually seen in the interiors of epithelioid cells, amastigotes were more frequently observed in the spleen of animals with granulomas. Hence, as observed in humans with long duration VL (30), these granulomas appear to reflect a persistent chronic inflammation in response to uncontrolled infection, rather than protection against the disease. Although granulomas may constitute a step towards infection control, the conditions in their interior may be subverted by a parasite to favour its survival and growth (31). In fact, interlukin-10 and other cytokines produced by granuloma cells may provide conditions for the survival of *Leishmania* (12) and other microorganisms (32,33). The fact that granulomas were only present in animals with positive spleen culture in our study supports the view that these granulomas may reflect a state of inefficient control of *Leishmania* infection by the host immune system.

The increase in the plasma cell population in lymphoid organs is a common finding in leishmaniasis (7,27,34), and it may be present at relatively early stages of the disease (35). In all of the infected groups examined in this study, a significant increase in the plasma cell population was observed to replace other cell populations in areas of the red pulp. A progressive replacement of T-cell areas in the white pulp was also observed in some animals (data not shown). The determinants of the changes in number or distribution of plasma cells in VL are poorly understood. However, in visceral leishmaniasis, several factors like the well-characterized polyclonal B-cell activation (36,37) and production of cytokines [e.g. IFN-γ, IL10 (38–41), and IL6 (42,43)] and chemokines (e.g. CXCL12) may contribute to plasma cell differentiation and retention in the red pulp (44). Further studies are necessary to define the mechanisms involved in the plasma cell replacement of normal cell populations in the spleen, and future work should also examine the potential contribution of cell population replacement to both the increase of polyclonal immunoglobulin levels observed in VL (45) and disease development.

Lymphoid hyperplasia, atrophy, and disorganization were all present in the infected dogs examined in this study. Hyperplasia was common in animals from the infected and potentially resistant to VL group, and atrophy and lymphoid tissue disorganization were most frequently present in the animals from the infected and potentially susceptible to VL group. Hyperplasia followed by atrophy of the lymphoid follicles may occur in the spleen during the course of VL (7,34,35). However, such atrophy and hyperplasia are not uniform in all compartments of the white pulp. As shown in Figure 2, marginal zone hyperplasia may occur with a variable degree of follicle atrophy. This finding, together with the effacement of the boundaries between usually distinct areas of the lymphoid tissue, indicates that structural disorganization is present in the white pulp, as suggested by Veress *et al.* (1977). The loss of the architectural structure of splenic tissue has been the subject of recent studies performed in mice infected with *Leishmania* (20) or other pathogens (21). A variety of changes, including the loss of cell populations and the impairment of cell migration to the different splenic compartments, have been observed in murine models of visceral leishmaniasis induced by TNF and IL-10 (20,46,47). We also observed that disorganization of the splenic lymphoid tissue was more frequent in animals with emaciation, even for those animals without evidence of *Leishmania* infection, than it was in animals without evidence of undernourishment. In these emaciated animals, weight loss may result from insufficient access to food, which, in its turn, may be associated with other infections (9,48,49). For instance, studies on experimental models have shown that viral infections may also cause splenic lymphoid tissue disorganization (21). A factor common to many infections, such as high levels of TNF (unfortunately not tested in this study), may account both for emaciation and for lymphoid tissue disorganization (20,50). Nevertheless, the fact that the intensity of white pulp disorganization was higher in animals with splenic parasitism than in those without indicates that an active *Leishmania* infection may play a causal role in a spleen disorganization process. Further studies are necessary to clarify the mechanisms involved in the structural disorganization of the splenic tissue in canine leishmaniasis and assess the potential role of such splenic changes in mediating increased susceptibility to bacterial infections in the late stages of the disease.

In conclusion, our study shows that natural infection by *L. chagasi* is associated with splenic architecture disruption, which is characterized by disorganization of normal lymphoid tissue, loss of normal spleen leucocyte diversity via replacement of leucocytes by plasma cells, and eventual atrophy of the lymphoid tissue. Studies examining the redistribution of the main cell populations, the profile of cytokine expression in the spleen, and the potential association of splenic alterations with increased susceptibility to bacterial infection in these animals are currently underway.

## References

[1] Desjeux P (2001). The increase in risk factors for leishmaniasis worldwide. Trans R Soc Trop Med Hyg.

[2] Badaro R, Jones TC, Lorenco R (1986). A prospective study of visceral leishmaniasis in an endemic area of Brazil. J Infect Dis.

[3] Caldas AJ, Silva DR, Pereira CC (2001). [*Leishmania* (*Leishmania*) *chagasi* infection in children from an endemic area of visceral leishmaniasis in the Sao Luis Island-MA, Brazil]. Rev Soc Bras Med Trop.

[4] Pozio E, Gradoni L, Bettini S, Gramiccia M (1981). Leishmaniasis in Tuscany (Italy). VI. Canine leishmaniasis in the focus of Monte Argentario (Grosseto). Acta Trop.

[5] Lanotte G, Rioux JA, Perieres J, Vollhardt Y (1979). [Ecology of leishmaniasis in the south of France. 10. Developmental stages and clinical characterization of canine leishmaniasis in relation to epidemiology. (author's transl)]. Ann Parasitol Hum Comp.

[6] Seaman J, Mercer AJ, Sondorp E (1996). The epidemic of visceral leishmaniasis in western Upper Nile, southern Sudan: course and impact from. 1984 to 1994. Int J Epidemiol.

[7] Veress B, Omer A, Satir AA, El Hassan AM (1977). Morphology of the spleen and lymph nodes in fatal visceral leishmaniasis. Immunology.

[8] Andrade TM, Carvalho EM, Rocha H (1990). Bacterial infections in patients with visceral leishmaniasis. J Infect Dis.

[9] Barrouin-Melo SM, Larangeira DF, de Andrade Filho FA (2006). Can spleen aspirations be safely used for the parasitological diagnosis of canine visceral leishmaniosis? A study on assymptomatic and polysymptomatic animals. Vet J.

[10] Ciaramella P, Oliva G, Luna RD (1997). A retrospective clinical study of canine leishmaniasis in 150 dogs naturally infected by *Leishmania infantum*. Vet Rec.

[11] Carrion J, Nieto A, Iborra S (2006). Immunohistological features of visceral leishmaniasis in BALB/c mice. Parasite Immunol.

[12] Wilson ME, Sandor M, Blum AM (1996). Local suppression of IFN-γ in hepatic granulomas correlates with tissue-specific replication of *Leishmania chagasi*. J Immunol.

[13] Barbosa Junior AA, Andrade ZA, Reed SG (1987). The pathology of experimental visceral leishmaniasis in resistant and susceptible lines of inbred mice. Braz J Med Biol Res.

[14] Paranhos-Silva M, Oliveira GG, Reis EA (2003). A follow-up of Beagle dogs intradermally infected with *Leishmania chagasi* in the presence or absence of sand fly saliva. Vet Parasitol.

[15] Werneck GL, Costa CH, Walker AM, David JR, Wand M, Maguire JH (2007). Multilevel modelling of the incidence of visceral leishmaniasis in Teresina, Brazil. Epidemiol Infect.

[16] Dos-Santos WL, Jesus EE, Paranhos-Silva M (2008). Associations among immunological, parasitological and clinical parameters in canine visceral leishmaniasis: Emaciation, spleen parasitism, specific antibodies and leishmanin skin test reaction. Vet Immunol Immunopathol.

[17] Sundar S, Jha TK, Thakur CP (2002). Oral miltefosine for Indian visceral leishmaniasis. N Engl J Med.

[18] Mebius RE, Kraal G (2005). Structure and function of the spleen. Nat Rev Immunol.

[19] Balogh P, Horvath G, Szakal AK (2004). Immunoarchitecture of distinct reticular fibroblastic domains in the white pulp of mouse spleen. J Histochem Cytochem.

[20] Engwerda CR, Ato M, Cotterell SE (2002). A role for tumor necrosis factor-α in remodeling the splenic marginal zone during *Leishmania donovani* infection. Am J Pathol.

[21] Benedict CA, De Trez C, Schneider K, Ha S, Patterson G, Ware CF (2006). Specific remodeling of splenic architecture by cytomegalovirus. Plos Pathog.

[22] Paranhos-Silva M, Pontes-de-Carvalho LC, de Sa Oliveira GG, Nascimento EG, dos-Santos WL (2001). Skin reactions to thimerosal and *Leishmania* in dogs from a leishmaniasis endemic area: it is better to keep them apart. Mem Inst Oswaldo Cruz.

[23] Baleeiro CO, Paranhos-Silva M, Dos Santos JC (2006). Montenegro's skin reactions and antibodies against different *Leishmania* species in dogs from a visceral leishmaniosis endemic area. Vet Parasitol.

[24] Verlag GF (1989). Cytological Basis of Immune Funcitons of the Spleen. Progrhistochem Cytochem.

[25] Glantz SA (1997). Primer of Bio-Statistics.

[26] Tafuri WL, Barbosa AJ, Michalick MS (1996). Histopathology and immunocytochemical study of type 3 and type 4 complement receptors in the liver and spleen of dogs naturally and experimentally infected with *Leishmania* (*Leishmania*) *chagasi*. Rev Inst Med Trop Sao Paulo.

[27] Tryphonas L, Zawidzka Z, Bernard MA, Janzen EA (1977). Visceral leishmaniasis in a dog: clinical, hematological and pathological observations. Can J Comp Med.

[28] Bertho AL, Santiago MA, Coutinho SG (1994). An experimental model of the production of metastases in murine cutaneous leishmaniasis. J Parasitol.

[29] dos-Santos WL, David J, Badaro R, de-Freitas LA (2004). Association between skin parasitism and a granulomatous inflammatory pattern in canine visceral leishmaniosis. Parasitol Res.

[30] Daneshbod K (1972). Visceral leishmaniasis (kala-azar) in Iran: a pathologic and electron microscopic study. Am J Clin Pathol.

[31] Murray HW (2001). Tissue granuloma structure-function in experimental visceral leishmaniasis. Int J Exp Pathol.

[32] Fenhalls G, Stevens L, Bezuidenhout J (2002). Distribution of IFN-γ, IL-4 and TNF-α protein and CD8 T cells producing IL-12p40 mRNA in human lung tuberculous granulomas. Immunology.

[33] Mariano M (1995). The experimental granuloma. A hypothesis to explain the persistence of the lesion. Rev Inst Med Trop Sao Paulo.

[34] Keenan CM, Hendricks LD, Lightner L, Johnson AJ (1984). Visceral leishmaniasis in the German shepherd dog. II. Pathology. Vet Pathol.

[35] Veress B, Abdalla RE, El Hassan AM (1983). Visceral spreading depletion of thymus-dependent regions and amyloidosis in mice and hamsters infected intradermally with *Leishmania* isolated from Sudanese cutaneous leishmaniasis. Br J Exp Pathol.

[36] Galvao-Castro B, Sa Ferreira JA, Marzochi KF, Marzochi MC, Coutinho SG, Lambert PH (1984). Polyclonal B cell activation, circulating immune complexes and autoimmunity in human american visceral leishmaniasis. Clin Exp Immunol.

[37] Campos-Neto A, Bunn-Moreno MM (1982). Polyclonal B cell activation in hamsters infected with parasites of the genus *Leishmania*. Infect Immun.

[38] Steiniger B, van der Meide PH (1993). High-dose interferon-γ alters the distribution of B lymphocytes and macrophages in rat spleen and lymph nodes. Immunology.

[39] Choe J, Choi YS (1998). IL-10 interrupts memory B cell expansion in the germinal center by inducing differentiation into plasma cells. Eur J Immunol.

[40] Caldas A, Favali C, Aquino D (2005). Balance of IL-10 and interferon-γ plasma levels in human visceral leishmaniasis: implications in the pathogenesis. BMC Infect Dis.

[41] Lage RS, Oliveira GC, Busek SU (2007). Analysis of the cytokine profile in spleen cells from dogs naturally infected by *Leishmania chagasi*. Vet Immunol Immunopathol.

[42] de Lima VM, Peiro JR, de Oliveira Vasconcelos R (2007). IL-6 and TNF-α production during active canine visceral leishmaniasis. Vet Immunol Immunopathol.

[43] Poeck H, Wagner M, Battiany J (2004). Plasmacytoid dendritic cells, antigen, and CpG-C license human B cells for plasma cell differentiation and immunoglobulin production in the absence of T-cell help. Blood.

[44] Hargreaves DC, Hyman PL, Lu TT (2001). A coordinated change in chemokine responsiveness guides plasma cell movements. J Exp Med.

[45] Manz RA, Arce S, Cassese G, Hauser AE, Hiepe F, Radbruch A (2002). Humoral immunity and long-lived plasma cells. Curr Opin Immunol.

[46] Ato M, Stager S, Engwerda CR, Kaye PM (2002). Defective CCR7 expression on dendritic cells contributes to the development of visceral leishmaniasis. Nat Immunol.

[47] Smelt SC, Engwerda CR, McCrossen M, Kaye PM (1997). Destruction of follicular dendritic cells during chronic visceral leishmaniasis. J Immunol.

[48] Andreotti R, Oliveira JM, Silva EA, Oshiro LM, Matos MD (2005). Occurrence of *Neospora caninum* in dogs and its correlation with visceral leishmaniasis in the urban area of Campo Grande, Mato Grosso do Sul, Brazil. Vet Parasitol.

[49] Trapp SM, Dagnone AS, Vidotto O, Freire RL, Amude AM, de Morais HS (2006). Seroepidemiology of canine babesiosis and ehrlichiosis in a hospital population. Vet Parasitol.

[50] Pearson RD, Cox G, Evans T, Smith DL, Weidel D, Castracane J (1990). Wasting and macrophage production of tumor necrosis factor/cachectin and interleukin 1 in experimental visceral leishmaniasis. Am J Trop Med Hyg.

